# Early adulthood weight change, midlife “Life’s essential 8” health status and risk of cardiometabolic diseases: a chinese nationwide cohort study

**DOI:** 10.1186/s12986-023-00765-w

**Published:** 2023-11-01

**Authors:** Qiuyu Cao, Mian Li, Guijun Qin, Li Yan, Jiang He, Min Xu, Yu Xu, Tiange Wang, Yuhong Chen, Shuangyuan Wang, Hong Lin, Zhiyun Zhao, Zhengnan Gao, Tianshu Zeng, Ruying Hu, Xuefeng Yu, Gang Chen, Qing Su, Yiming Mu, Lulu Chen, Xulei Tang, Qin Wan, Guixia Wang, Feixia Shen, Zuojie Luo, Yingfen Qin, Li Chen, Yanan Huo, Qiang Li, Zhen Ye, Yinfei Zhang, Chao Liu, Youmin Wang, Shengli Wu, Tao Yang, Huacong Deng, Jiajun Zhao, Lixin Shi, Guang Ning, Weiqing Wang, Jieli Lu, Yufang Bi

**Affiliations:** 1 Department of Endocrine and Metabolic Diseases, Shanghai Institute of Endocrine and Metabolic Diseases, Ruijin Hospital, Shanghai Jiao Tong University School of Medicine, 197 Rui-Jin 2nd Road, Shanghai, 200025 China; 2 Shanghai National Clinical Research Center for metabolic Diseases, Key Laboratory for Endocrine and Metabolic Diseases of the National Health Commission of the PR China, Shanghai Key Laboratory for Endocrine Tumor, Ruijin Hospital, Shanghai Jiao Tong University School of Medicine, Shanghai, China; 3 The First Affiliated Hospital of Zhengzhou University, Zhengzhou, China; 4 Sun Yat-sen Memorial Hospital, Sun Yat-sen University, Guangzhou, China; 5 Department of Epidemiology, Tulane University School of Public Health and Tropical Medicine, New Orleans, USA; 6 Dalian Municipal Central Hospital, Dalian, China; 7 Union Hospital, Tongji Medical College, Huazhong University of Science and Technology, Wuhan, China; 8 Zhejiang Provincial Center for Disease Control and Prevention, Hangzhou, China; 9 Tongji Hospital, Tongji Medical College, Huazhong University of Science and Technology, Wuhan, China; 10 Fujian Provincial Hospital, Fujian Medical University, Fuzhou, China; 11 Xinhua Hospital, Shanghai Jiao Tong University School of Medicine, Shanghai, China; 12 Chinese people’s Liberation Army General Hospital, Beijing, China; 13 The First Hospital of Lanzhou University, Lanzhou, China; 14 The Affiliated Hospital of Southwest Medical University, Luzhou, China; 15 The First Hospital of Jilin University, Changchun, China; 16 The First Affiliated Hospital of Wenzhou Medical University, Wenzhou, China; 17 The First Affiliated Hospital of Guangxi Medical University, Nanning, China; 18 Qilu Hospital of Shandong University, Jinan, China; 19 Jiangxi Provincial People’s Hospital Affiliated to Nanchang University, Nanchang, China; 20 The Second Affiliated Hospital of Harbin Medical University, Harbin, China; 21 Central Hospital of Shanghai Jiading District, Shanghai, China; 22 Jiangsu Province Hospital on Integration of Chinese and Western Medicine, Nanjing, China; 23 The First Affiliated Hospital of Anhui Medical University, Hefei, China; 24 Karamay Municipal People’s Hospital, Xinjiang, China; 25 The First Affiliated Hospital of Nanjing Medical University, Nanjing, China; 26 The First Affiliated Hospital of Chongqing Medical University, Chongqing, China; 27 Shandong Provincial Hospital Affiliated to Shandong University, Jinan, China; 28 Guiqian International General Hospital, Guiyang, China

**Keywords:** Weight, Cardiovascular health, Cardiovascular disease, Diabetes

## Abstract

**Background:**

The association between weight change during early adulthood and cardiometabolic diseases remains uncertain in Chinese population. Whether the association varies with comprehensive cardiovascular health (CVH) in midlife assessed by “Life’s Essential 8” has not been characterized. We aim to examine the associations of early adulthood weight change and midlife “Life’s Essential 8” CVH status with cardiometabolic outcomes in a Chinese cohort.

**Methods:**

The study participants were from the China Cardiometabolic Disease and Cancer Cohort (4 C) Study. This analysis included 72,610 middle-aged and older participants followed for a median of 3.6 years. At baseline, the participants recalled body weight at age 20 and 40 years, and we calculated change in weight and BMI between 20 and 40 years of age. Health behaviors information in “Life’s Essential 8” was collected by questionnaire, and health factors were measured in the study center. During follow-up, we ascertained incident cardiovascular events based on medical records, and diagnosed incident diabetes according to the American Diabetes Association 2010 criteria.

**Results:**

72,610 study participants were included with a mean age of 56.0 ± 8.8 years and 29% of them were males. Weight gain of more than 10 kg between 20 and 40 years of age was associated with 22% increased risk of incident cardiovascular events (HR: 1.22; 95%CI: 1.04–1.43) and 38% increased risk of diabetes (HR: 1.38; 95%CI: 1.25–1.53) compared to stable weight. Besides, the association of weight gain more than 10 kg in early adulthood with cardiometabolic risk was even stronger in those with low CVH score in midlife (HR: 2.44; 95%CI: 2.01–2.97 for incident cardiovascular events; HR: 2.20; 95%CI: 1.90–2.55 for incident diabetes) or with few ideal cardiovascular health metrics in midlife.

**Conclusions:**

Our study indicated that weight gain in early adulthood was associated with significantly increased risk of cardiometabolic diseases. And the association could be stronger in those with poor CVH profiles in midlife. These findings confirmed the significance of weight management during early adulthood and suggested that individuals who experienced substantial weight gain in early life should be encouraged to maintain good CVH status in Chinese population.

**Supplementary Information:**

The online version contains supplementary material available at 10.1186/s12986-023-00765-w.

## Background

Obesity has been a great threat to global health, with a rapidly increasing prevalence in the past decade worldwide [1]. The increasing rate in China was even faster, with an estimated 85 million adults aged 18 to 69 years in China who were obese in 2018, which was 3 times as many as in 2004 [2, 3]. Mounting evidence demonstrated that excess adiposity was an indispensable risk factor for cardiometabolic disease.

Excess adiposity tends to accrue during early and middle adulthood for most people [4]. Adult weight gain has been proved to associate with an increased risk of type 2 diabetes and cardiovascular diseases (CVD) [5–7]. However, the results from previous studies were not entirely consistent, and evidence of association between long-term weight change during early and middle adulthood and cardiometabolic disease was still insufficient. Moreover, evidence from Asian or Chinese population was even more limited, with most studies performed in Western populations. Over the past four decades, China has witnessed a substantial rise in obesity along with rapid economic development and urbanization [8]. And most of the current middle-aged and older Chinese adults experienced substantial weight change between 20 and 40 years of age, possibly due to transitions from principally active lifestyles and calorie-restricted diets to sedentary lifestyles and western diets [9]. Therefore, delineation of the relationship between weight change in early adulthood and health outcomes is crucial in the Chinese population.

Previous studies found that the association between body mass index (BMI) and mortality could be significantly influenced by lifestyle behaviors [10]. A prospective study of National Health and Nutrition Examination Survey (NHANES) also demonstrated that later inadequate physical activity worsened the negative impact of early-adulthood weight gain [11]. But the association of comprehensive lifestyle and metabolic factors and weight change with cardiometabolic disease is unclear. Recently, the American Heart Association (AHA) proposed “Life’s Essential 8”, including 8 health behaviors and biochemical factors to quantify a composite score for measuring, monitoring, and modifying cardiovascular health (CVH) [12]. It is of great significance to uncover the association of weight change in early adulthood and CVH status in midlife with incident cardiometabolic disease, which could provide guidance to prevention of CVD and diabetes through long-term weight management and behavioral modification.

To address these knowledge gaps, using data from a nationwide, prospective Chinese cohort, we aimed to examine the relation of weight change in young adulthood and midlife “Life’s Essential 8” health status with incident CVD events and diabetes.

## Methods

### Study design and participants

The study participants were from the China Cardiometabolic Disease and Cancer Cohort (4 C) Study, which is a nationwide population-based prospective cohort study consisting of community-dwelling adults aged 40 years or older [13]. The study protocol and informed consent were approved by the Committee on Human Research at Ruijin Hospital affiliated to Shanghai Jiao Tong University School of Medicine, Shanghai, China. All participants provided the written informed consent.

The details of the 4 C Study design have been described previously [14, 15]. The baseline survey was conducted between 2011 and 2012. 193,846 participants were recruited from local resident registration systems of 20 community sites representing 16 provincial administrative regions of mainland China. Eligible men and women aged ≥ 40 years were identified from local resident registration systems. Trained community health workers visited eligible individual’s homes and invited them to participate in the study. We intended to enroll both men and women, but more women participated in the study, possibly because women care more about their health and have more flexible schedule due to home working, compared to men. The follow-up visit was conducted between 2014 and 2016, and 170,240 participants (87.8%) attended. As shown in Supplemental Fig. 1, for the current analyses, we excluded 84,167 individuals with missing data on weight at age 20 years or 40 years, and 260 participants whose height, weight or BMI at 20 or 40 years was outside the valid range (height > 200 cm or height < 120 cm or weight > 150 kg or weight < 20 kg or BMI < 13 kg/m^2^ or BMI > 50 kg/m^2^), and 12,203 individuals with missing data on any metric of cardiovascular health. A total of 72,610 participants were included in the analyses. For the analyses of incident CVD events, we further excluded 1,326 participants with prior CVD events at baseline and 11,736 participants with missing data on incident CVD events during follow-up, leaving 59,548 participants. And for analyses of incident diabetes, we excluded 17,868 diabetes patients at baseline and 7,934 participants with missing data on incident diabetes during follow-up, leaving 46,808 participants. We compared the key baseline characteristics of the study participants and participants who were excluded from the analysis in Supplemental Table 1.

### Data collection

At baseline and follow-up visits, data collection was performed in local community clinics by trained staff following standardized protocols. Standard questionnaires were administered to collect information on demographic characteristics, education level, family history of CVD events and diabetes, medical history and lifestyle habits. Skilled nurses measured blood pressure according to standard protocols. Blood samples were collected after overnight fasting. Then a standard oral glucose tolerance test (OGTT) was conducted, and 2-hour post-load blood specimens were collected. Fasting and 2-hour post-load plasma glucose were measured at local hospitals. Serum samples were shipped in dry ice to the central laboratory, where lipids profiles and creatinine were measured using an autoanalyzer (Abbott Laboratories, IL). Glycated hemoglobin (HbA1c) was tested using finger capillary whole blood using high-performance liquid chromatography (VARIANT™ II Systems, BIO-RAD, Hercules, CA, USA) at the central laboratory.

### Assessments of weight change

Data on weight at age 20 years and 40 years were recalled at the baseline survey. Baseline height and body weight were measured according to a standard protocol. BMI at age 20 and 40 years was calculated as self-reported weight (kg) divided by squared height (m^2^) at baseline.

Absolute weight change over 20 kg was redefined to 20 kg. We classified absolute weight change from age 20 to 40 years into five groups: weight loss > 2.5 kg, weight loss ≤ 2.5 kg or gain < 2.5 kg (reference group), weight gain between ≥ 2.5 kg and < 5.0 kg, weight gain between ≥ 5.0 kg and < 10.0 kg, and weight gain ≥ 10 kg. On the basis of a study by Chen et al [16], we also defined five BMI change patterns using BMI at age 20 and 40 years: stable normal pattern (< 23.0 at both times), maximum overweight pattern (23.0-24.9 at either time but not ≥ 25.0 at the other time), obese to non-obese pattern (≥ 25.0 at 20 years and < 25.0 at 40 years), non-obese to obese pattern (< 25.0 at 20 years and ≥ 25.0 at 40 years), and stable obesity (≥ 25.0 at both times). We used BMI cut points more suitable for Asian and Chinese in our study [17].

### Definition of Life’s essential 8 CVH score and status

According to the updated definition proposed by AHA in 2022 [12], we redefined and scored the baseline CVH score based on 8 components, including nicotine exposure, physical activity, diet, sleep, BMI, blood lipids, blood glucose, and blood pressure. Each of the 8 CVH metrics was scored from 0 to 100, with 100 points representing ideal cardiovascular health metrics (ICVHM)). The overall CVH score was calculated as the average of 8 component metric scores. Participants with overall CVH scores of 80–100 were considered to have high CVH, while moderate CVH referred to 50–79 and low CVH referred to 0–49. Detailed methods and scoring criteria referred to AHA Presidential Advisory were presented in Supplemental Table 2.

### Ascertainment of incident CVD events and diabetes

Information on CVD events was obtained from hospital records. Two members of the outcome adjudication committee, masked to the baseline characteristics, independently reviewed all medical material and defined CVD events as a composite of non-fatal myocardial infarction, non-fatal stroke, hospitalized or treated heart failure, and cardiovascular deaths. Discrepancies were resolved by discussion involving other members of the committee.

At the follow-up visit, we repeated OGTT and blood sample collection to assess fasting and 2-hour post-load plasma glucose, and HbA1c. Incident diabetes was diagnosed according to the ADA 2022 criteria [18]: fasting plasma glucose ≥ 126 mg/dL, or OGTT 2-hour post-load plasma glucose ≥ 200 mg/dL, or HbA1c ≥ 6.5%, or a diagnosis by physicians during follow-up.

### Statistical analysis

According to weight change categories from age 20 years to 40 years, baseline characteristics of participants were expressed as means with standard deviations (SD) for continuous variables and numbers with percentages for categorical variables.

The association of weight change categories and BMI change patterns with the risk of incident clinical outcomes was evaluated using Cox proportional hazards models to generate hazard ratio (HR) and 95% confidence interval (CI). In the crude model 1, we adjusted for age, sex and weight at age 20 years (only in weight change analysis). Model 2 further adjusted for education level, CVD events or diabetes family history. Model 3 fully adjusted for individual CVH metric scores, except for BMI score, based on model 2. We also depicted the association between weight change from age 20 years to 40 years and risk for outcomes by restricted cubic splines and calculated the non-linear P value, adjusting for covariates in model 3. Furthermore, we investigated the associations of weight change categories and overall CVH status with incident diabetes and CVD events in which we divided all participants into 13 categories as weight change within 2.5 kg and other weight change patterns with 3 degrees of CVH (high, moderate, and low). Besides, we estimated the associations of weight gain ≥ 10 kg and number of ICVHMs (without ideal BMI) with incident CVD events and diabetes.

Five sensitivity analyses were performed. Considering BMI in the CVH metrics may have significant effect on weight change analysis, a sensitivity analysis was performed to test the combined association of weight change categories and CVH status estimated by 7 metrics without BMI with outcomes (Supplemental Fig. 2). To test the impact of missingness and exclusion of participants on the results, we conducted multiple imputation by fully conditional specification and generated 10 imputed datasets [19], and the results were pooled by Rubin’s Rule. The results were shown in Supplemental Tables 4 and Supplemental Fig. 3. We also performed another complementary analysis to test the combined association of weight change categories and CVH status estimated by 7 metrics without glucose with diabetes (Supplemental Fig. 4). In addition, we evaluated the association between weight gain ≥ 10 kg and outcomes in subgroups of individual CVH metric and further tested their interactions by including the product term (exposure × stratification variable) in the models (Supplemental Fig. 5). We also evaluated the association between weight change and outcomes in females and males and tested their interactions (Supplemental Table 5).

We used SAS software (version 9.4), and R software (version 4.1.1) for statistical analyses. All reported P values are nominal. Statistical significance was a two-tailed P < 0.05.

## Results

### Baseline characteristics

We included 72,610 study participants with a mean age of 56.0 ± 8.8 years and 29% of them were males. As shown in Table 1, nearly one third of the participants gained weight ≥ 10 kg during age 20–40 years, and 28% of them gained weight 5–10 kg, but only 6.6% had weight loss > 2.5 kg in that time period. Participants who gained weight ≥ 10 kg were younger, higher-educated, had more families with CVD events or diabetes, and had lower CVH points, representing worse CVH status, than people who had weight change of -2.5 to 2.4 kg. 72.5% of people who gained weight ≥ 10 kg had moderate CVH, and 23.2% of them presented low CVH, but only 4.3% of them had high CVH. Participants who lost weight > 2.5 kg had similar demographic characteristics with those who had stable weight, but their CVH score was the highest among the 5 groups, and 12.6% of them had high CVH. The trend of individual CVH metric was consistent with the overall CVH.

**Table 1:** Baseline characteristics of participants with different weight change patterns

Baseline characteristics	Weight change patterns from age 20 years to 40 years				
	Weight loss > 2.5 kg	Weight loss ≤ 2.5 kg or gain < 2.5 kg	Weight gain between ≥ 2.5 kg and < 5.0 kg	Weight gain between ≥ 5.0 kg and < 10.0 kg	Weight gain ≥ 10.0 kg
No. of participants (n (%))	4,782 (6.6)	16,537 (22.8)	7,499 (10.3)	20,297 (28.0)	23,495 (32.4)
Age (years)	56.5 ± 8.6	58.0 ± 9.1	56.8 ± 8.9	56.3 ± 8.8	53.9 ± 8.3
Female (n (%))	3,635 (76.0)	11,657 (70.5)	5,443 (72.6)	14,919 (73.5)	15,909 (67.7)
Weight at baseline (kg)	55.6 ± 9.3	58.2 ± 9.5	60.0 ± 9.2	63.0 ± 9.4	69.6 ± 10.5
Weight in age 20 years (kg)	60.2 ± 8.9	55.1 ± 8.4	53.4 ± 7.9	53.1 ± 7.9	52.6 ± 8.3
Weight in age 40 years (kg)	54.1 ± 8.4	55.5 ± 8.3	56.9 ± 7.9	59.4 ± 7.9	67.4 ± 9.9
BMI at baseline (kg)	22.2 ± 3.2	23.2 ± 3.1	23.9 ± 3.1	24.9 ± 3.1	26.7 ± 3.4
BMI in age 20 years (kg/m^2^)	24.1 ± 3.0	22.0 ± 2.8	21.2 ± 2.6	20.9 ± 2.6	20.2 ± 2.7
BMI in age 40 years (kg/m^2^)	21.6 ± 2.8	22.1 ± 2.8	22.6 ± 2.6	23.4 ± 2.6	25.9 ± 3.2
Highschool attendance (n (%))	1,654 (34.6)	5,856 (35.4)	2,586 (34.5)	7,999 (39.4)	10,397 (44.3)
CVD family history (n (%))	670 (14.0)	2,232 (13.5)	1,021 (13.6)	3,170 (15.6)	4,059 (17.3)
Diabetes family history (n (%))	589 (12.3)	2,090 (12.6)	1,021 (13.6)	3,161 (15.6)	4,691 (20.0)
Overall CVH score	66.7 ± 11.6	64.1 ± 12.0	63.2 ± 11.8	61.7 ± 11.9	58.6 ± 12.0
Overall CVH status (n (%))					
High	600 (12.6)	1,647 (10.0)	602 (8.0)	1,374 (6.8)	1,009 (4.3)
Moderate	3,801 (79.5)	12,898 (78.0)	5,889 (78.5)	15,638 (77.1)	17,031 (72.5)
Low	381 (8.0)	1,992 (12.0)	1,008 (13.4)	3,285 (16.2)	5,455 (23.2)
Never smoking (n (%))	3,828 (80.1)	12,909 (78.1)	6,014 (80.2)	16,396 (80.8)	17,867 (76.1)
Ideal physical activity (n (%))	1,213 (25.4)	3,972 (24.0)	1,678 (22.4)	4,402 (21.7)	5,390 (22.9)
Ideal diet (n (%))	1,423 (29.8)	4,844 (29.3)	2,217 (29.6)	6,081 (30.0)	7,264 (30.9)
Ideal sleep health (n (%))	3,773 (78.9)	12,147 (79.5)	5,963 (79.5)	16,182 (79.7)	18,401 (78.3)
Ideal body weight (n (%))	3,099 (64.8)	8,462 (51.2)	3,035 (40.5)	5,636 (27.8)	2,698 (11.5)
Ideal blood pressure (n (%))	1,887 (39.5)	5,379 (32.5)	2,212 (29.5)	5,494 (27.1)	5,458 (23.2)
Ideal blood lipids (n (%))	2,492 (52.1)	7,666 (46.4)	3,346 (44.6)	8,488 (41.8)	9,130 (38.9)
Ideal blood glucose (n (%))	1,308 (27.4)	3,758 (22.7)	1,633 (21.8)	4,010 (19.8)	3,964 (16.9)

Continuous variables were shown by means ± standard deviation. Categorical variables were shown by number (percent). Definitions of cardiovascular health status, CVH score and ideal cardiovascular health metrics were consistent with “Life’s Essential 8”. CVH, cardiovascular health

### Association between weight change in early adulthood and CVD events or diabetes

Weight change in early adulthood was associated with incident CVD events and diabetes. After fully adjusting for age, sex, education level, weight at age 20 years, CVD family history (or diabetes family history) and baseline individual CVH score except for BMI score, gaining weight ≥ 10 kg was associated with a 22% higher risk of incident CVD events (HR: 1.22, 95%CI: 1.04–1.43) and a 38% higher risk of incident diabetes (HR: 1.38, 95%CI: 1.25–1.53) than weight change of -2.5 to 2.4 kg. And gaining weight 5.0 to 9.9 kg was related to a 12% higher risk of incident diabetes (HR: 1.12, 95%CI: 1.01–1.24) compared to remaining stable weight (Table 2). Besides, losing weight > 2.5 kg was associated with 26% increased risk of incident CVD event with borderline statistical significance (HR: 1.26, 95%CI: 0.99–1.61), and not associated with risk of diabetes (HR: 0.88, 95%CI: 0.74–1.05). According to the BMI change patterns, after full adjustment, change from non-obesity to obesity was associated with a 16% higher risk of incident CVD events (HR: 1.16, 95%CI: 1.00-1.34) and 47% higher risk of diabetes (HR: 1.47, 95%CI: 1.35–1.61) than maintaining normal BMI, and stable obesity was associated with a 23% higher risk of incident CVD events (HR: 1.23, 95%CI: 1.01–1.50) and 36% higher risk of diabetes (HR: 1.36, 95%CI: 1.18–1.56) than maintaining normal BMI. Changing from obesity to non-obesity was also associated with a 64% higher risk of incident CVD events (HR: 1.64, 95%CI: 1.14–2.35), but not associated with risk of diabetes (HR: 0.93, 95%CI: 0.68–1.26) (Supplemental Table 3). The results remained similar in the multiple imputed datasets (Supplemental Table 4).

**Table 2:** Risk for CVD events and diabetes related to weight change patterns

Outcomes	Model	Weight change between age 20 years and 40 years				
		Weight loss > 2.5 kg	Weight loss ≤ 2.5 kg or gain < 2.5 kg	Weight gain between ≥ 2.5 kg and < 5.0 kg	Weight gain between ≥ 5.0 kg and < 10.0 kg	Weight gain ≥ 10.0 kg
CVD events	Cases/ Total number	86 / 3,977	290 / 13,618	104 / 6,200	370 / 16,544	415 / 19,209
	CVD incidence	2.16%	2.13%	1.68%	2.24%	2.16%
	Hazard ratios (95%CI)					
	Model 1	1.13 (0.89–1.44)	1 [Reference]	0.92 (0.73–1.15)	1.28 (1.10–1.49)	1.52 (1.30–1.77)
	Model 2	1.14 (0.89–1.46)	1 [Reference]	0.92 (0.73–1.15)	1.27 (1.09–1.48)	1.49 (1.28–1.74)
	Model 3	1.26 (0.99–1.61)	1 [Reference]	0.86 (0.69–1.08)	1.14 (0.98–1.34)	1.22 (1.04–1.43)
Diabetes	Cases/ Total number	155 / 3,443	632 / 11,176	312 / 5,090	858 / 13,075	1179 / 14,024
	Diabetes incidence	4.50%	5.65%	6.13%	6.56%	8.41%
	Hazard ratios (95%CI)					
	Model 1	0.79 (0.66–0.95)	1 [Reference]	1.18 (1.03–1.35)	1.25 (1.13–1.39)	1.69 (1.53–1.86)
	Model 2	0.80 (0.67–0.95)	1 [Reference]	1.17 (1.02–1.34)	1.24 (1.12–1.38)	1.67 (1.51–1.84)
	Model 3	0.88 (0.74–1.05)	1 [Reference]	1.09 (0.95–1.25)	1.12 (1.01–1.24)	1.38 (1.25–1.53)

Model 1 adjusted for age, sex and weight at age 20 years; Model 2 further adjusted for education level, CVD family history (or diabetes family history) based on Model 1; Model 3 further adjusted for baseline smoking points, physical activity points, diet points, sleep points, blood pressure, blood glucose and blood lipids points based on Model 2. CVD, cardiovascular disease

**Fig. 1:**
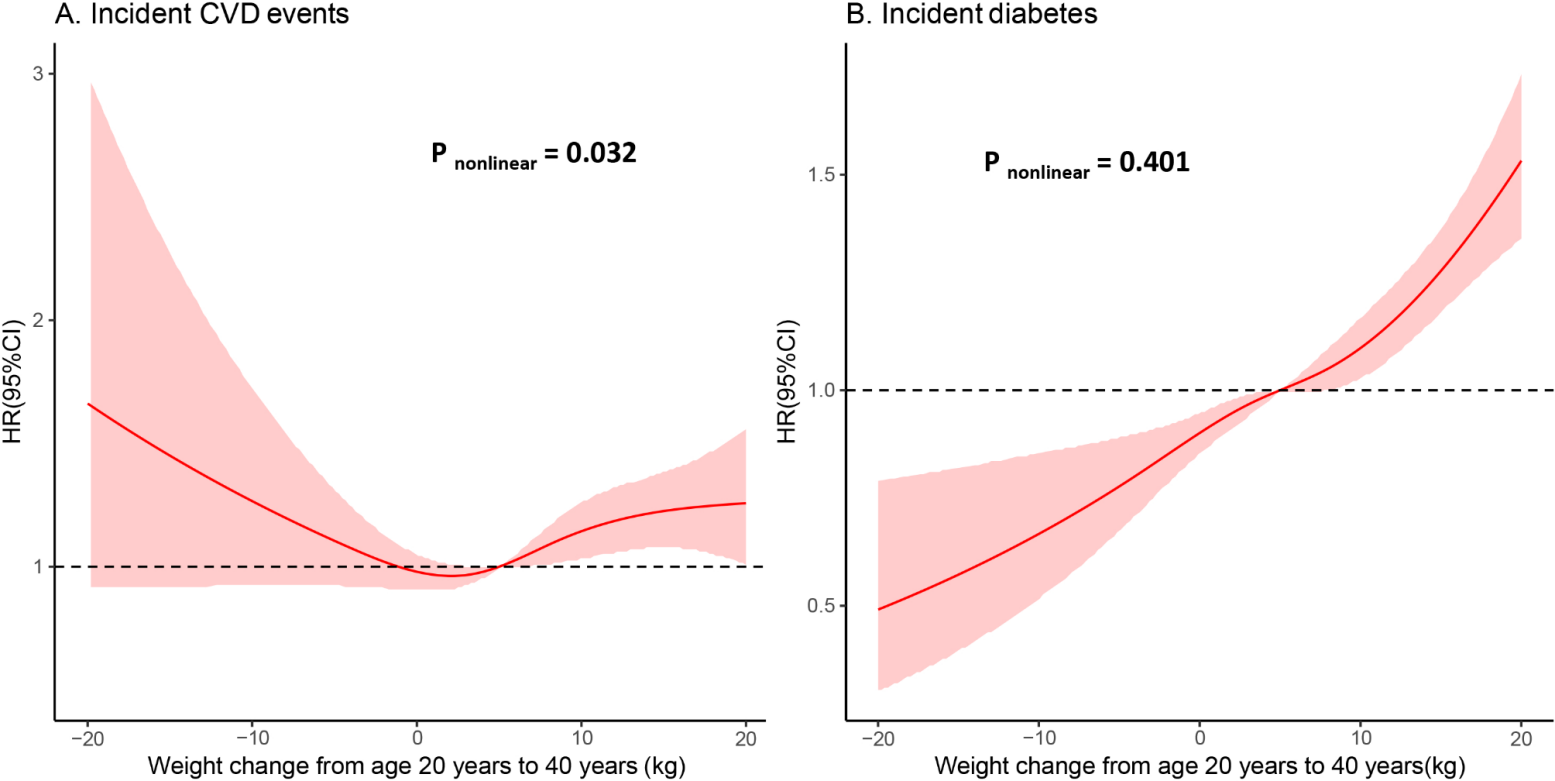
Association between weight change and risk for CVD or diabetes Model adjusted for age, sex, education level, weight at age 20 years, CVD family history (or diabetes family history), baseline smoking points, physical activity points, diet points, sleep points, blood pressure, blood glucose and blood lipids points. CVD, cardiovascular disease

As shown in Fig. 1, a U-shaped relationship between weight change from age 20 to 40 years and risk for CVD events was observed (P for nonlinearity = 0.032), while a linear relationship was detected between weight change and risk for incident diabetes (P for nonlinearity = 0.401).

### Associations of midlife CVH status and early adulthood weight change with the risk of CVD events and diabetes

**Fig. 2:**
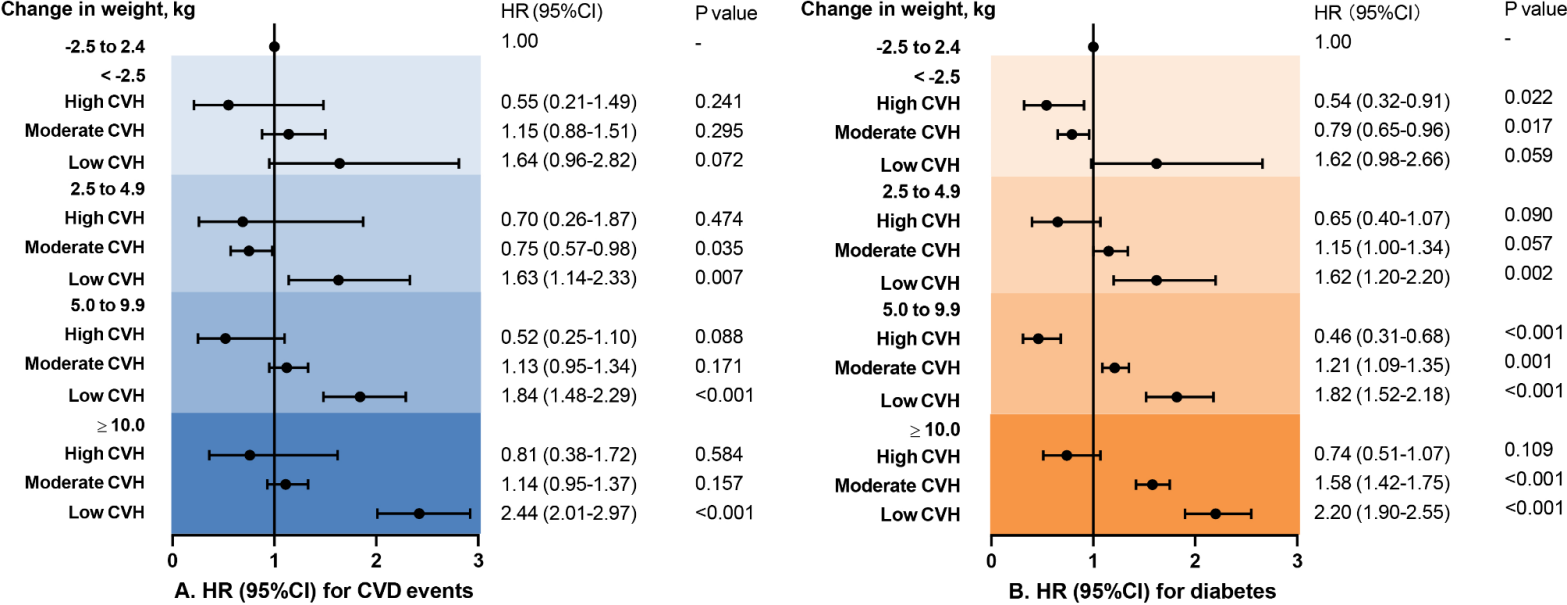
Associations of weight change, CVH level with risk for CVD or diabetes The baseline CVH level was evaluated by “Life’s Essential 8” components, including BMI. Model adjusted for age, sex, education level, weight at age 20 years, CVD family history (or diabetes family history). BMI, body mass index; CVD, cardiovascular disease; CVH, cardiovascular health

**Fig. 3:**
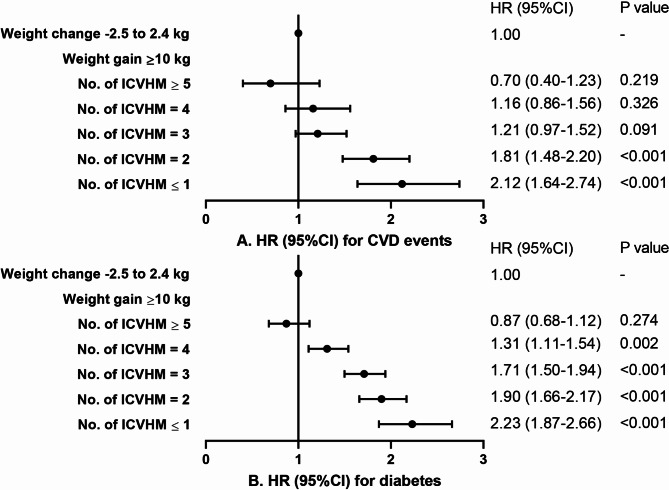
Associations of weight change, ICVHM number with risk for CVD or diabetes The ideal cardiovascular health metrics were 7 components of “Life’s Essential 8”, without ideal BMI. Model adjusted for age, sex, education level, weight at age 20 years, CVD family history (or diabetes family history). BMI, body mass index; CVD, cardiovascular disease; ICVHM, ideal cardiovascular health metric

The associations between weight change and CVD events or diabetes varied with overall CVH status. As presented in Fig. 2, in those who had high or moderate CVH in midlife, gaining weight in early adulthood was not related to an increased risk of CVD events (HR: 0.81, 95%CI: 0.38–1.72), while in those who had low CVH in midlife, gaining weight was associated with an increased risk of incident CVD events. The risk of CVD events increased by 144% (HR: 2.44, 95%CI: 2.01–2.97) in those who gained weight over 10 kg in early life and had low CVH in midlife compared to people with stable weight in early adulthood. Furthermore, in those who had high CVH in midlife, weight change in age 20–40 years was not associated with an increased risk of diabetes than stable weight (HR: 0.74, 95%CI: 0.51–1.07), but in those who had moderate or low CVH score in midlife, the risk of diabetes increased gradually with early adulthood weight gain. Participants with weight gain over 10 kg and low CVH had a 120% increased risk of developing diabetes (HR: 2.20, 95%CI: 1.90–2.55) compared to those with stable weight. The results remained similar in sensitivity analyses (Supplemental Figs. 2–4). Furthermore, we found that the risk of cardiovascular events and diabetes associated with weight gain in age 20–40 years increased gradually with decreasing number of ICVHMs in midlife. In those with 1 or less ICVHMs in midlife, weight gain over 10 kg in early adulthood was associated with 112% higher risk of CVD events (HR: 2.12, 95%CI: 1.64–2.74) and 123% higher risk of diabetes (HR: 2.23, 95%CI: 1.87–2.66) compared to stable weight (Fig. 3).

We observed no interaction between sex and weight change patterns on risk of outcomes, with P for interaction 0.368 for CVD and 0.306 for diabetes (Supplemental Table 5). Limited interaction between individual CVH metric in midlife and weight change patterns on risk of outcomes was observed, with most Ps for interaction > 0.05 (Supplemental Fig. 5). However, midlife blood lipid had significant interaction with early adulthood weight change on the risk of CVD events (P for interaction = 0.035), and the risk associated with weight gain was higher in those with non-ideal blood lipid.

## Discussion

In this large-scale prospective study in China, we demonstrated that weight gain in age 20–40 years was associated with increased risk of incident CVD events and diabetes compared to stable weight. In addition, the association between early adulthood weight change and cardiometabolic outcomes was much stronger in those who had low CVH score or few ideal CVH metrics in midlife. These findings suggested the significance of weight management during early adulthood and the potential benefit of achieving good CVH status in those who experienced substantial weight gain in early life.

Most previous studies investigating the association between weight change patterns and risk of cardiovascular diseases were conducted in European and American populations, few have been performed in Asian populations where people generally have lower BMI levels but more adipose body composition [20, 21]. And the findings in Asian populations were inconsistent. The Japan Public Health Center-based prospective study measured weight change of participants between baseline and 5-year follow up visit in their late adulthood, and over 60% of the participants had a stable weight (weight change within 2.5 kg) during that period. The authors found that CVD mortality risk tended to increase with weight loss both in men and women, whereas its increase with weight gain was observed only in women [22]. Besides, the Singapore Chinese Health Study observed weight change of participants during 6 years of follow-up in their late adulthood and found that both weight gain and loss conferred excess CVD mortality risk in middle-aged and elderly Chinese [23]. However, in our baseline analysis conducted recently, a weight loss of 2.5 kg during early adulthood conferred a lower risk of CVD compared with stable weight [24]. The heterogeneity of conclusions may be due to the difference of study design, definition of weight change periods, ascertainment of CVD outcomes and statistical methods. Most of these studies collected weight data over a short period of time in late adulthood, when the participants could have less weight variation compared to early adulthood. So, the proportion of participants with substantial weight gain was much lower than that in our cohort study. Besides, these studies only adjusted for limited lifestyle and metabolic covariates [22, 23], and our previous study used self-reported CVD history as the outcome, which could cause recall bias [24].

Our nationwide prospective study used weight data of participants in age 20 and 40 years to identify the weight change pattern in their early adulthood, which is also a period of rapid economic development in China and could lead to substantial lifestyle transition and weight variation in Chinese population. The participants who gained weight more than 10 kg in early adulthood were younger and had higher education level, which was also consistent with the socioeconomic background. We observed that weight gain more than 10 kg in early adulthood was associated with incremental risk of incident CVD events after fully adjustment of socioeconomic, lifestyle and metabolic risk factors. Although weight loss was not significantly associated with CVD events, changing from obesity to non-obesity was associated with 64% increment of CVD risk compared to stable normal BMI, which indicated that the risk of CVD events was related to both weight change and initial weight. Our finding on weight loss is consistent with a previous study in the US National Health and Nutrition Examination Survey (NHANES) which observed that weight loss from middle to late adulthood was associated with an increased risk of all-cause mortality and CVD mortality [16]. These findings confirmed the significance of maintaining normal weight (avoiding significant weight gain or weight loss) in early adulthood to prevent cardiovascular events.

Our study also found that weight gain in early adulthood was associated with increased diabetes risk, which was consistent with previous evidence [25]. But we didn’t observe decreased risk of diabetes in those who lost weight between age 20–40 years. Results from diabetes prevention trials suggested that weight loss through lifestyle modification could prevent or delay diabetes [26]. It was possible that adjustment for lifestyle and metabolic factors attenuated the association between weight loss and diabetes.

The “Life’s Essential 8” CVH approach proposed by AHA recently was designed to comprehensively evaluate the status of cardiovascular health. It added evaluation of sleep habits to the conventional “Life’s Simple 7” CVH metrics [27]. Previous studies suggested that later lifestyle habits, including physical activity and smoking might modify the association between early life weight change and mortality [11, 28]. Our previous research also observed that the negative effect of early-life famine exposure on diabetes could be modified by ideal CVH metrics [29]. Nevertheless, evidence of the effect of updated CVH status on association between early adulthood weight change and cardiometabolic diseases was still required. Our study demonstrated that the risk of CVD events and diabetes increased gradually with worsening midlife CVH status in each stratum of early adulthood weight change. Those who achieved good CVH status in midlife avoided increased risk associated with early adulthood weight gain, and those who had poor CVH level in midlife and gained weight over 10 kg in early adulthood showed the highest risk. Besides, the association of weight change with cardiometabolic diseases could change with number of ICVHMs. We only observed an interaction between blood lipids level and weight change. Weight change was associated with lipoprotein particle concentration and particle size [30], but the underlying mechanism still requires more evidence. Our findings indicated that the association between early adulthood weight change and cardiometabolic diseases might variate with composite CVH status in midlife, and implied the importance of a healthy lifestyle and ideal metabolic traits in the prevention of cardiometabolic disease among individuals who have experienced substantial weight gain.

The strengths of this study include a nationwide prospective cohort design, the large sample size, and the detailed information about lifestyle factors.

Our study also has several limitations. Firstly, weight at age 20 and 40 years was self-reported, and recall bias might be possible. However, previous validation studies have shown high accuracy of self-reported weight [31]. Secondly, we only collected lifestyle and metabolic information at baseline, and data of time-varying CVH metrics from age 20 years was not available, which requires further investigation. Thirdly, the study participants were only followed for a mean of 3.6 years. This relatively short follow-up duration reduced the number of incident CVD events and diabetes and the study’s statistical power. Fourthly, we could not differentiate intentional and unintentional weight change due to lack of data. Given the observational nature of our study, currently no reliable causal relationship could be established. Thus, future randomized controlled studies are warranted. Finally, the pregnancy weight and postpartum weight changes data were unavailable in our study. Since female participants are in the reproductive age period in early to middle adulthood, and previous studies suggested that the pregnancy weight and postpartum weight change could be associated with risks of CVD and diabetes [32, 33], the mechanism behind the association warrants further investigation.

## Conclusions

In conclusion, we found that weight gain in early adulthood increased risk of CVD events and diabetes in later life. This association could become stronger in those who had poor CVH status in midlife. Our findings emphasize the importance of weight maintenance in early adulthood and suggest that individuals who experienced substantial weight gain in early life should be encouraged to maintain good CVH status for prevention of cardiometabolic diseases in Chinese population.

### Electronic supplementary material

Below is the link to the electronic supplementary material.

Supplementary Material 1

## Data Availability

The data are available from the corresponding authors upon reasonable request.

## Abbreviations

4 C: China Cardiometabolic Disease and Cancer Cohort
AHA: American Heart Association
BMI: body mass index
CVD: cardiovascular disease
CVH: cardiovascular health
ICVHM: ideal cardiovascular health metric
