# Supplementary material for: Early adulthood weight change, midlife “Life’s essential 8” health status and risk of cardiometabolic diseases: a chinese nationwide cohort study
Source: Nutr Metab (Lond). 2023 Nov 1;20:48. doi: 10.1186/s12986-023-00765-w (PMC10621175; doi:10.1186/s12986-023-00765-w)
Supplement: Supplementary file 1 — Supplementary Material 1 [file 12986_2023_765_MOESM1_ESM.pdf]

Supplement material

Supplemental Table 1. Baseline Characteristics of the Study Participants and Participants Who were Excluded from the Analysis

Characteristic	Study Participants	Excluded participants*
Number of participants	72610	97630
Age, years	56.0 ± 8.8	57.7 ± 9.3
Male sex, n (%)	21047 (29.0)	37783 (38.7)
CVD family history, n (%)	11152 (15.4)	13245 (13.6)
BMI, kg/m^2	24.8 ± 3.6	24.7 ± 3.7
FPG, mg/dl	108.0 ± 29.3	107.4 ± 30.5
2h PPG, mg/dl	150.3 ± 69.2	149.2 ± 71.5
HbA1c, %	6.06 ± 1.03	6.01 ± 1.05
Diabetes family history, n (%)	11552 (15.9)	9949 (10.2)
SBP, mmHg	131.1 ± 19.8	135.8 ± 21.5
DBP, mmHg	78.0 ± 10.8	79.1 ± 11.5
Non-HDL cholesterol, mg/dl	139.3 ± 40.3	143.1 ± 39.1

Values are mean (SD) for continuous variables and number (proportion) for categorical variables. *Participants excluded from the analysis = Participants who were successfully followed up – Participants included in the analysis for weight change and cardiovascular health status = Participants with missing data on weight of age 20 and 40 years, weight out of range, or any missing CVH metric = 170240 – 72610 = 97630. Please refer to Figure S1 for a flowchart of the study participants.

FPG: fasting plasma glucose; 2h PPG: 2h post-load plasma glucose; HbA1c: glycated hemoglobin; BMI: body mass index; SBP: systolic blood pressure; DBP: diastolic blood pressure; HDL: high-density lipoprotein; SD: standard deviation.

Supplemental Table 2. Definition of Life's Essential 8 CVH score and status

CVH metric	Method of measurement	Quantification of CVH metrics
Nicotine exposure	Self-reported use of cigarettes	Points Tobacco use of secondhand smoke exposure<br>100: Never smoker<br>75: Former smoker, quit ≥5 years<br>50: Former smoker, quit 1–<5 years<br>25: Former smoker, quit <1 year<br>0: Current smoker<br>Subtract 20 points (unless score is 0) for living with active indoor smoker in home
Physical activity	Self-reported physical activity	Points Minutes of moderate or vigorous physical activity per week<br>100: ≥150<br>90: 120–149<br>80: 90–119<br>60: 60–89<br>40: 30–59<br>20: 1–29<br>0: 0
Diet	Self-reported healthy diet of 7 components*	Points Number of healthy diet components<br>100: 6 or 7<br>80: 5 or 4<br>50: 3<br>25: 2<br>0: 0 or 1
Sleep health	Self-reported average hours of sleep per night	Points Hours of sleep per night<br>100: 7-<9<br>90: 9-<10<br>70: 6-<7<br>40: 5-<6 or ≥10<br>20: 4–<5<br>0: <4
BMI	Objective measurement of weight and height	Points BMI (kg/m^2)<br>100: <23.0<br>75: 23.0-<25.0<br>50: 25.0-<30.0<br>25: 30.0-<35.0<br>0: ≥35.0
Blood lipids	Fasting Plasma non–HDL cholesterol tested at the central laboratory	Points Non-HDL cholesterol (mg/dl)<br>100: <130<br>60: 130-159<br>40: 160-189<br>20: 190-219

		0: ≥220<br>Subtract 20 points if treated level
Blood glucose	Fasting plasma glucose, 2h post-load plasma glucose tested locally, and Fasting HbA1c tested at the central laboratory	Points Glycemic status<br>100: NGR: No history of diabetes and FPG<100 mg/dl and OGTT-2h PPG<140mg/dl and HbA1c<5.7%<br>60: Prediabetes: No diabetes and (FPG 100-125mg/dl or OGTT-2h PPG 140-199mg/dl or HbA1c 5.7-6.4%)<br>40: Diabetes with HbA1c<7.0%<br>30: Diabetes with HbA1c: 7.0-7.9%<br>20: Diabetes with HbA1c: 8.0-8.9%<br>10: Diabetes with HbA1c: 9.0-9.9%<br>0: Diabetes with HbA1c: ≥10.0%
Blood pressures	Objective measurement of SBP and DBP	Points SBP and DBP (mmHg)<br>100: <120/80 (optimal)<br>75: 120-129/<80 (elevated)<br>50: 130-139 or 80-89 (stage 1 hypertension)<br>25: 140-159 or 90-99<br>0: ≥160 or ≥100<br>Subtract 20 points if treated level

*The healthy diet score included the following 7 components: fruits and vegetables ≥4.5 cups/day, fish ≥two 3.5-oz servings/week, livestock and poultry meat: 40-75g/day, sweets/sugar-sweetened beverages ≤450 kcal/week, soy protein ≥25 g/day, milk or yogurt ≥3 cups/week, alcohol ≤ 2 servings per day for men or ≤ 1 serving per day for women.

CVH: cardiovascular health; BMI: body mass index; HDL: high-density lipoprotein; NGR: normal glucose regulation; FPG: fasting plasma glucose; OGTT: oral glucose tolerant test; PPG: post-load plasma glucose; HbA1c: glycated hemoglobin; SBP: systolic blood pressure; DBP: diastolic blood pressure.

Supplemental Table 3. Hazard ratios for incident CVD events and diabetes related to BMI change patterns from age 20 years to 40 years

Outcomes	Model	BMI change patterns from age 20 years to 40 years				
		Obese to non-obese (n=1212)	Stable normal (n=30087)	Maximum overweight (n=18873)	Non-obese to obese (n=17553)	Stable Obese (n=4885)
CVD events	Model 1	1.59 (1.11-2.28)	1.00	1.24 (1.08-1.43)	1.45 (1.26-1.68)	1.51 (1.24-1.84)
	Model 2	1.61 (1.13-2.31)	1.00	1.24 (1.07-1.43)	1.44 (1.24-1.66)	1.51 (1.24-1.83)
	Model 3	1.64 (1.14-2.35)	1.00	1.12 (0.98-1.30)	1.16 (1.00-1.34)	1.23 (1.01-1.50)
Diabetes	Model 1	0.89 (0.65-1.21)	1.00	1.18 (1.08-1.30)	1.78 (1.63-1.94)	1.57 (1.36-1.81)
	Model 2	0.88 (0.65-1.21)	1.00	1.17 (1.07-1.28)	1.76 (1.61-1.92)	1.55 (1.34-1.79)
	Model 3	0.93 (0.68-1.26)	1.00	1.08 (0.99-1.19)	1.47 (1.35-1.61)	1.36 (1.18-1.56)

Model 1 adjusted for age and sex; Model 2 further adjusted for education level, CVD family history (or diabetes family history) based on Model 1; Model 3 further adjusted for baseline smoking points, physical activity points, diet points, sleep points, blood pressure, blood glucose and blood lipids points based on Model 2.

Supplemental Table 4. Hazard ratios for incident CVD events and diabetes related to weight change patterns from age 20 years to 40 years in the imputed datasets

	Model	Weight change between age 20 years and 40 years				
		Weight loss > 2.5 kg	Weight loss ≤ 2.5 kg or gain < 2.5 kg	Weight gain between ≥ 2.5 kg and < 5.0 kg	Weight gain between ≥ 5.0 kg and < 10.0 kg	Weight gain ≥ 10.0 kg
CVD events	Model 1	1.02 (0.90-1.17)	1.00	1.10 (0.98-1.23)	1.19 (1.08-1.31)	1.40 (1.25-1.56)
	Model 2	1.01 (0.89-1.16)	1.00	1.09 (0.97-1.22)	1.19 (1.08-1.31)	1.40 (1.25-1.56)
	Model 3	1.08 (0.94-1.24)	1.00	1.04 (0.92-1.16)	1.09 (0.99-1.20)	1.18 (1.06-1.32)
Diabetes	Model 1	0.82 (0.72-0.93)	1.00	1.11 (1.00-1.23)	1.26 (1.17-1.36)	1.59 (1.47-1.72)
	Model 2	0.82 (0.71-0.93)	1.00	1.10 (1.00-1.22)	1.26 (1.16-1.35)	1.57 (1.45-1.70)
	Model 3	0.87 (0.76-0.99)	1.00	1.07 (0.97-1.18)	1.17 (1.09-1.27)	1.38 (1.27-1.50)

Model 1 adjusted for age and sex and weight at age 20 years; Model 2 further adjusted for education level, CVD family history (or diabetes family history) based on Model 1; Model 3 further adjusted for baseline smoking points, physical activity points, diet points, sleep points, blood pressure, blood glucose and blood lipids points based on Model 2.

Supplemental Table 5. Hazard ratios for incident CVD events and diabetes related to weight change patterns from age 20 years to 40 years in males and females

	Sex	Model	Weight change between age 20 years and 40 years					P for interaction
			Weight loss > 2.5 kg	Weight loss ≤ 2.5 kg or gain < 2.5 kg	Weight gain between ≥ 2.5 kg and < 5.0 kg	Weight gain between ≥ 5.0 kg and < 10.0 kg	Weight gain ≥ 10.0 kg	
CVD events	Male	Model 1	1.08 (0.71-1.65)	1.00	0.66 (0.44-0.99)	1.17 (0.91-1.50)	1.33 (1.04-1.71)	0.368
		Model 2	1.13 (0.74-1.72)	1.00	0.67 (0.44-1.01)	1.15 (0.90-1.48)	1.29 (1.01-1.66)	
		Model 3	1.18 (0.77-1.81)	1.00	0.63 (0.42-0.96)	1.03 (0.80-1.32)	1.02 (0.79-1.32)	
	Female	Model 1	1.17 (0.86-1.58)	1.00	1.09 (0.83-1.43)	1.37 (1.12-1.66)	1.65 (1.35-2.01)	
		Model 2	1.17 (0.86-1.58)	1.00	1.08 (0.82-1.42)	1.35 (1.11-1.64)	1.62 (1.33-1.98)	
		Model 3	1.31 (0.97-1.78)	1.00	1.01 (0.77-1.33)	1.23 (1.01-1.50)	1.35 (1.11-1.66)	
Diabetes	Male	Model 1	0.73 (0.50-1.05)	1.00	1.10 (0.86-1.41)	1.25 (1.04-1.51)	1.72 (1.45-2.05)	0.306
		Model 2	0.73 (0.51-1.05)	1.00	1.10 (0.85-1.41)	1.25 (1.04-1.51)	1.71 (1.43-2.04)	
		Model 3	0.78 (0.54-1.13)	1.00	1.02 (0.80-1.32)	1.14 (0.94-1.37)	1.43 (1.20-1.71)	
	Female	Model 1	0.81 (0.66-0.99)	1.00	1.21 (1.03-1.42)	1.25 (1.10-1.42)	1.66 (1.47-1.87)	
		Model 2	0.81 (0.66-1.00)	1.00	1.20 (1.02-1.41)	1.24 (1.10-1.41)	1.64 (1.45-1.85)	
		Model 3	0.92 (0.75-1.12)	1.00	1.12 (0.95-1.32)	1.11 (0.98-1.26)	1.36 (1.20-1.54)	

Model 1 adjusted for age and weight at age 20 years; Model 2 further adjusted for education level, CVD family history (or diabetes family history) based on Model 1; Model 3 further adjusted for baseline smoking points, physical activity points, diet points, sleep points, blood pressure, blood glucose and blood lipids points based on Model 2.

Supplemental Figure 1. Participant flow

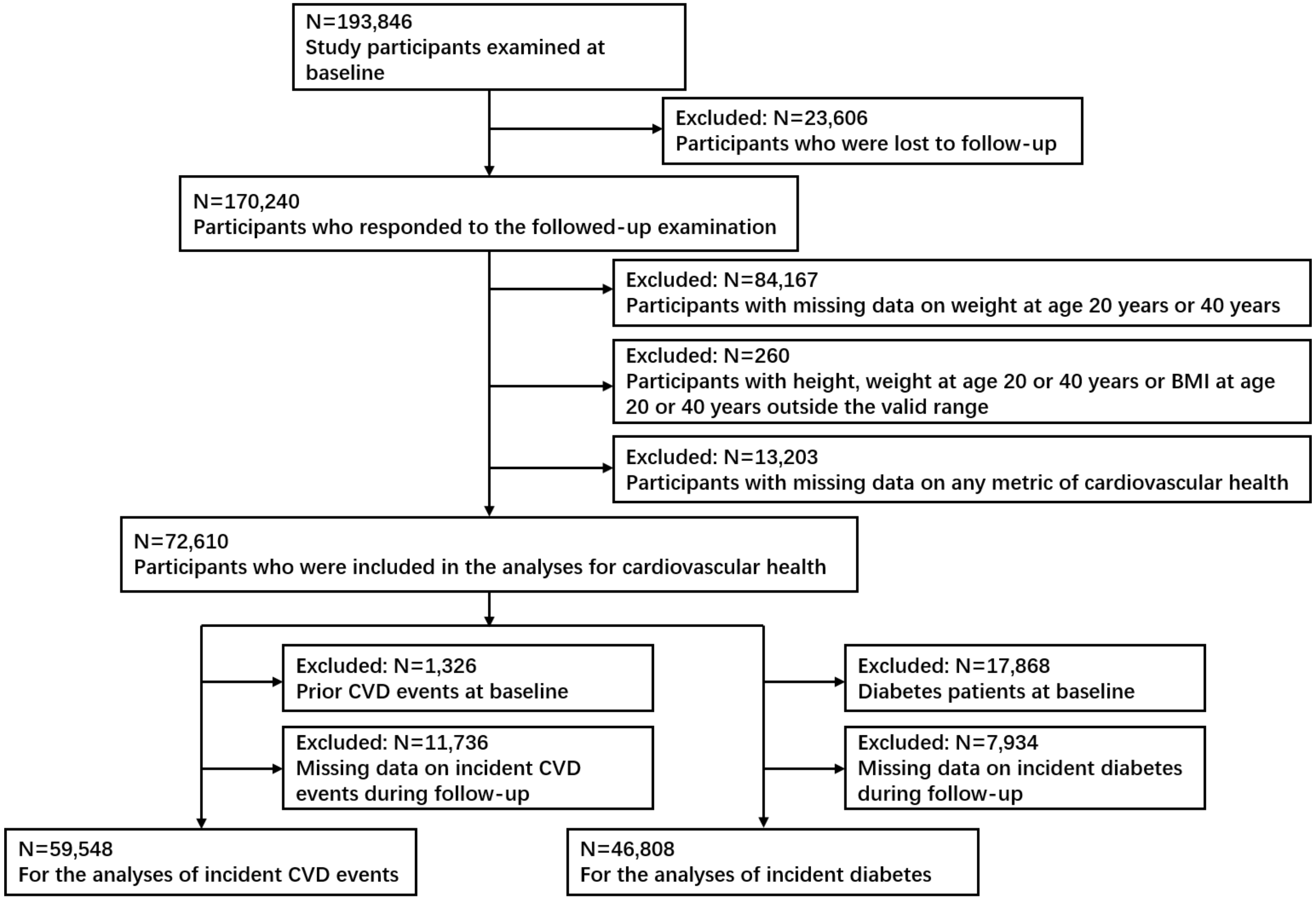

Supplemental Figure 2. Risk of CVD events and diabetes according to combination of weight change and CVH level categories (without BMI points)

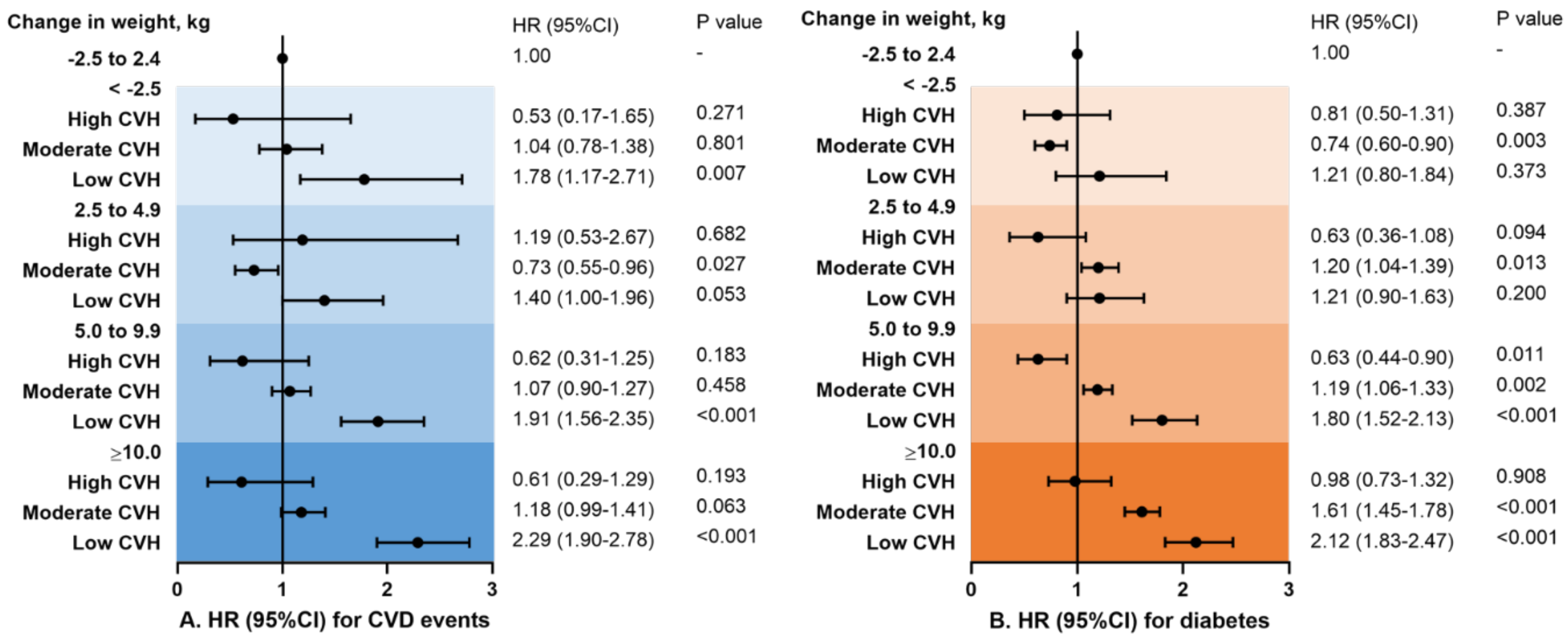

Model adjusted for age, sex, education level, weight at age 20 years, CVD family history (or diabetes family history).

Supplemental Figure 3. Risk of CVD events and diabetes according to combination of weight change and CVH level categories in the imputed datasets

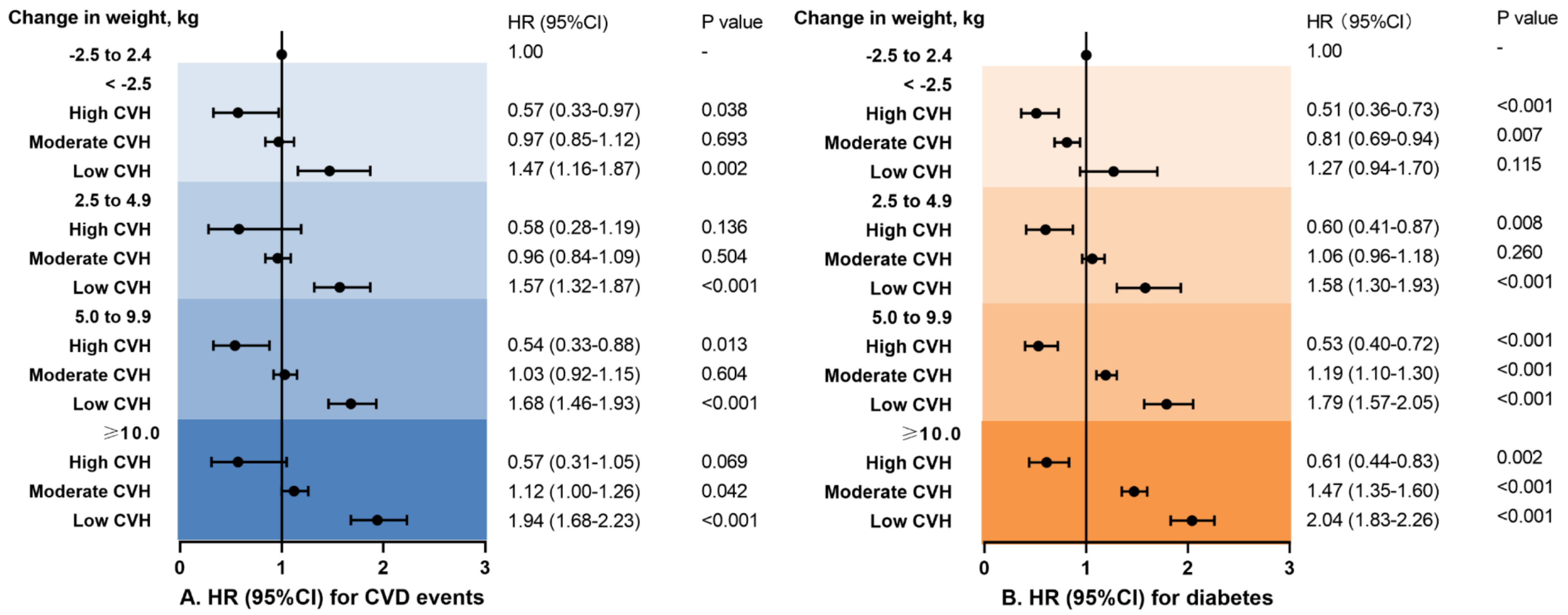

Model adjusted for age, sex, education level, weight at age 20 years, CVD family history (or diabetes family history).

Supplemental Figure 4. Risk of diabetes according to combination of weight change and CVH level categories (without glucose points)

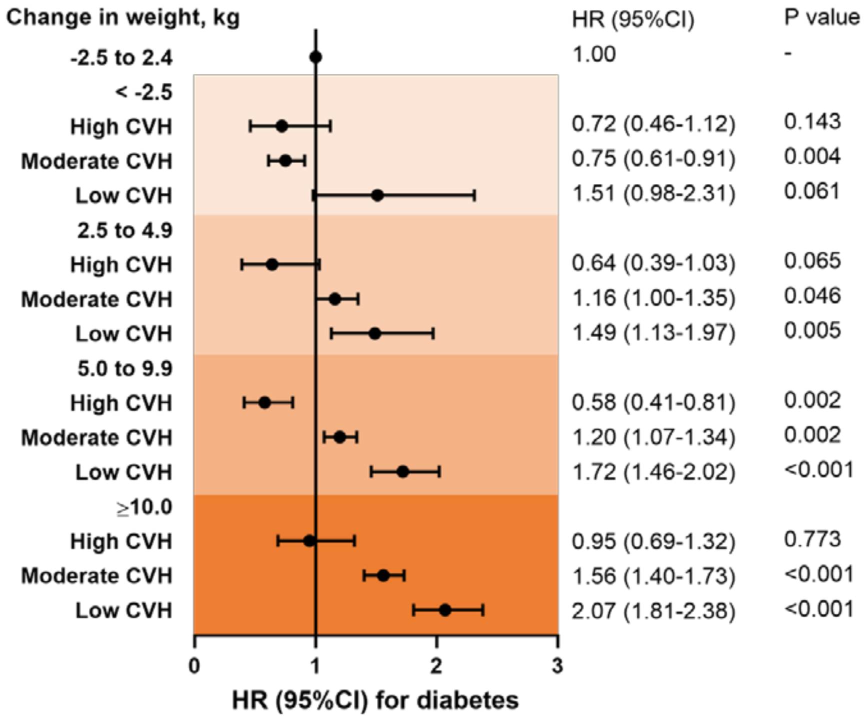

Model adjusted for age, sex, education level, weight at age 20 years and diabetes family history.

Supplemental Figure 5. Association between weight change and CVD events or diabetes in individual CVH metric subgroups

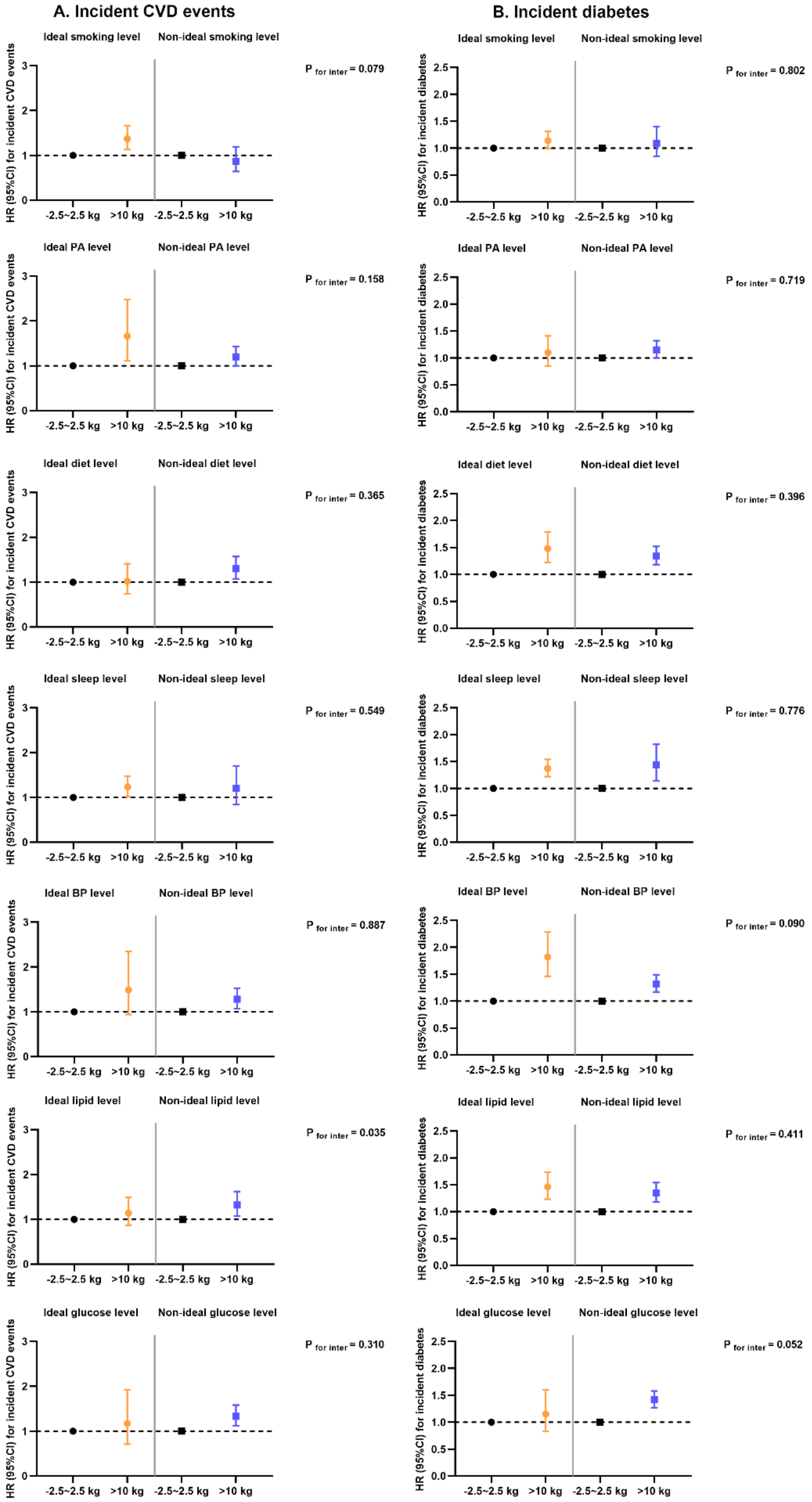

Model adjusted for age, sex, education level, weight at age 20 years, CVD family history, diabetes family history and other CVH metrics points.
